# Supplementary figures and images for: LEET: stock market forecast with long-term emotional change enhanced temporal model
Source: PeerJ Comput Sci. 2024 Apr 2;10:e1969. doi: 10.7717/peerj-cs.1969 (PMC11041952; doi:10.7717/peerj-cs.1969)

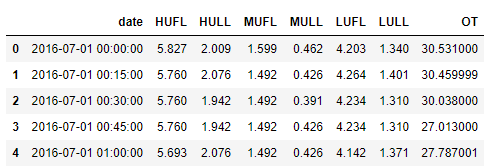

Supplement: Supplemental Information 1 [file peerj-cs-10-1969-s001.zip › Romember_model/rope_informer/img/data.png]

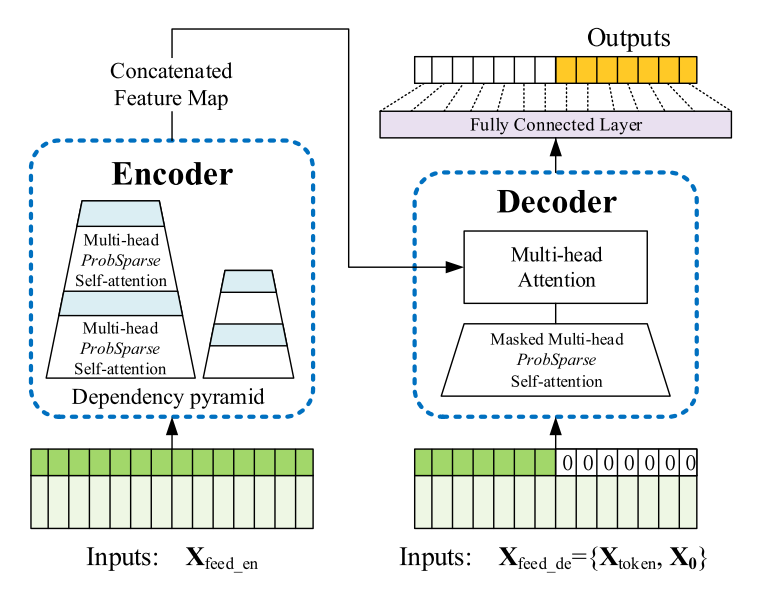

Supplement: Supplemental Information 1 [file peerj-cs-10-1969-s001.zip › Romember_model/rope_informer/img/informer.png]

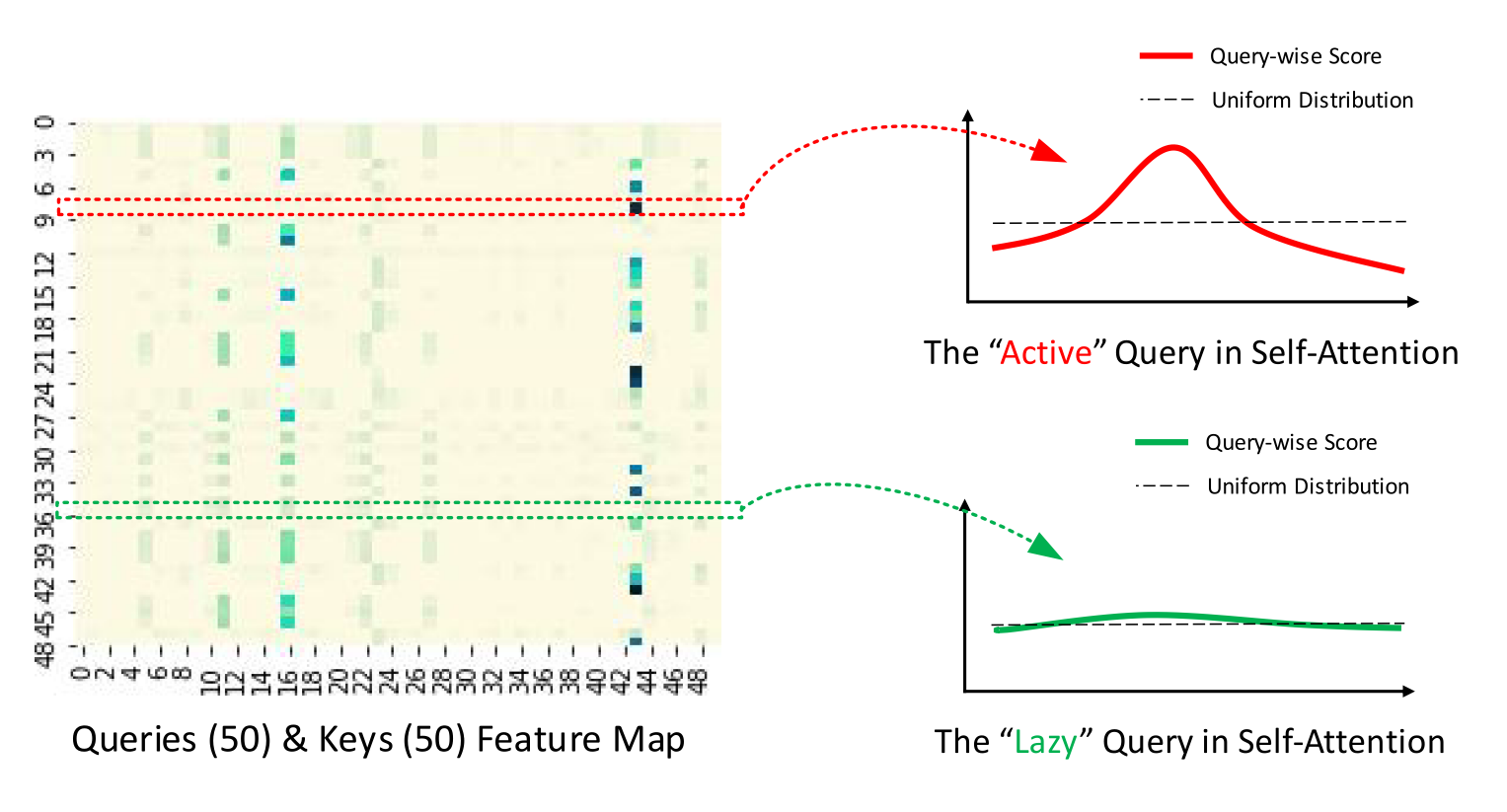

Supplement: Supplemental Information 1 [file peerj-cs-10-1969-s001.zip › Romember_model/rope_informer/img/probsparse_intro.png]

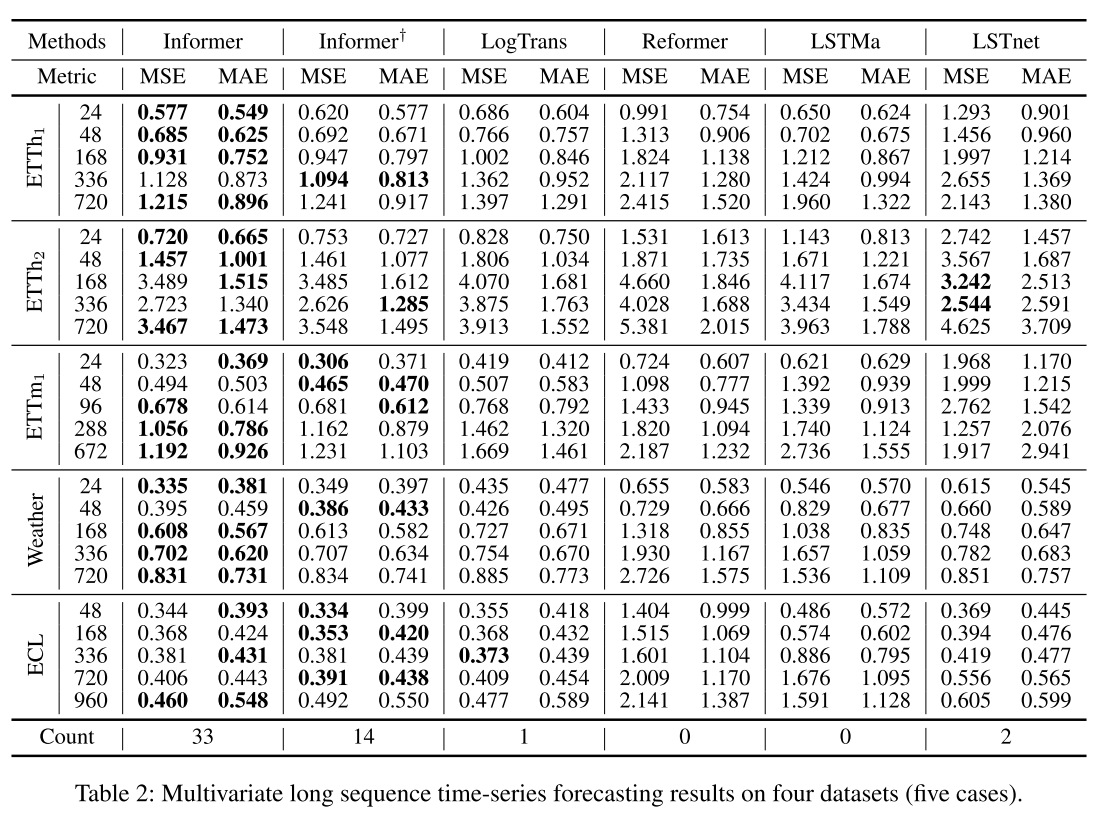

Supplement: Supplemental Information 1 [file peerj-cs-10-1969-s001.zip › Romember_model/rope_informer/img/result_multivariate.png]

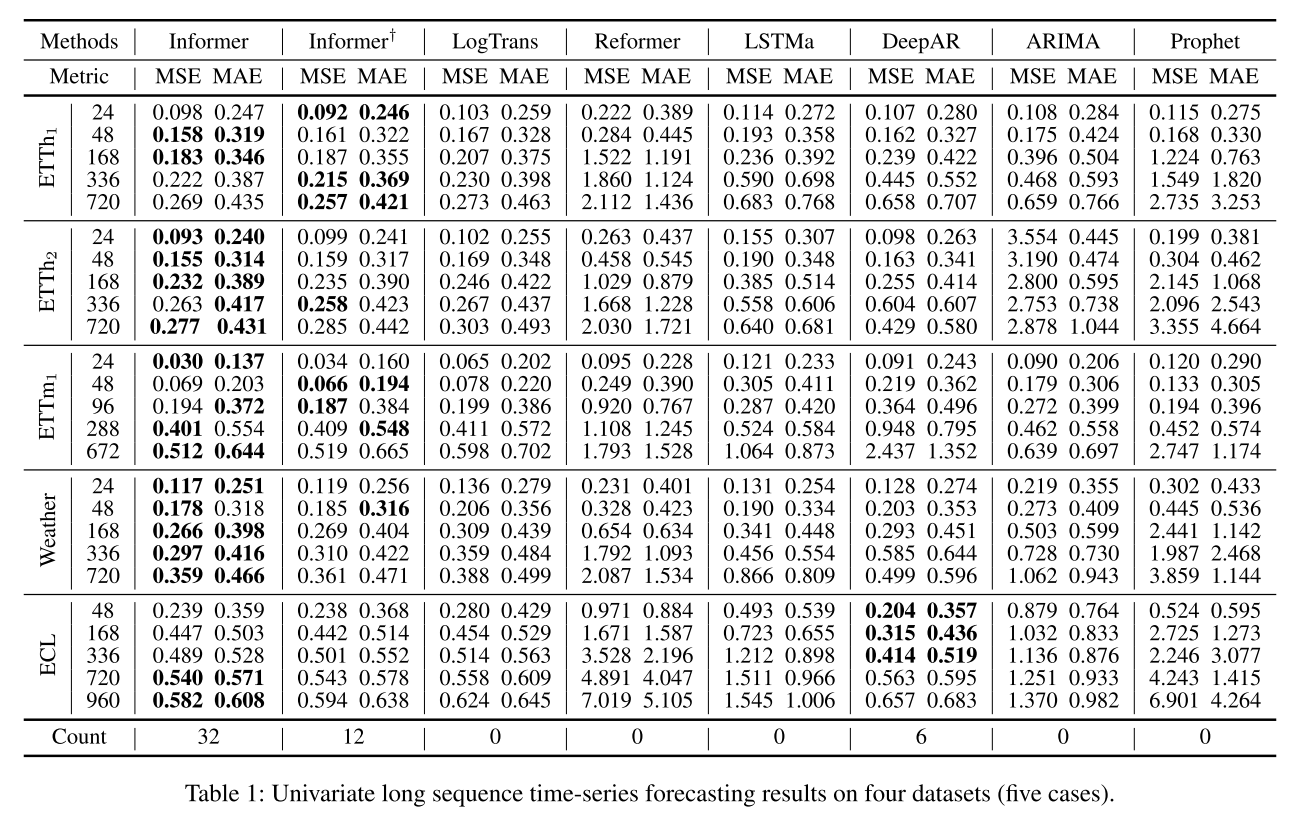

Supplement: Supplemental Information 1 [file peerj-cs-10-1969-s001.zip › Romember_model/rope_informer/img/result_univariate.png]
